# Would It Be Better if Instructors Technically Adjust Their Image or Voice in Online Courses? Impact of the Way of Instructor Presence on Online Learning

**DOI:** 10.3389/fpsyg.2021.746857

**Published:** 2021-09-21

**Authors:** Mingze Yuan, Jialing Zeng, Aihua Wang, Junjie Shang

**Affiliations:** ^1^Lab of Learning Sciences, Graduate School of Education, Peking University, Beijing, China; ^2^Department of Human Development, Teachers College, Columbia University, New York, NY, United States

**Keywords:** online education, video courses, instructor presence, image and voice, online learners

## Abstract

This study investigated the effects of the changes in the image and voice of instructors in online video courses on online learner's learning achievement, social presence, learning satisfaction, and academic emotion. Two simultaneous online experiments were conducted with 122 college students in the image experiment, where the course videos varied in terms of the instructor's image (original image, face-beautified image, virtual image, and no image), and 93 college students in the voice experiment, where the course videos varied in terms of the instructor's voice (original voice, mutated voice, computer-synthesized voice). The results showed that learners viewing videos without instructor images had better learning achievements and less academic boredom relative to those who viewed videos with instructor images. However, the real instructor images were able to promote learners' learning satisfaction of instructor-student interaction more than no image and virtual image and promote satisfaction of instructor teaching more than virtual image. Meanwhile, learners' evaluation of the real instructor images was better than that of the virtual instructor image, and their evaluation of the face-beautified instructor image was better than the original image. Moreover, learners evaluated real instructor voices better than the computer-synthesized voice. In addition, the linear regression analysis revealed that the evaluations of both instructor's image and voice had a positive relationship with learners' social presence, learning satisfaction, and enjoyment, whereas they had a negative relationship with learner's boredom. And the evaluation of the instructor's image positively predicted student's transfer learning achievement. Thus, we suggested that the way of instructor presence should be well-designed and integrated with the course's instructional design and image and voice processing technology can be applied to assist online video course development.

## Introduction

Since the outbreak of COVID-19 in 2020, online education has been strongly promoted worldwide, as it provides highly convenient, flexible, and commonly shared online resources for students. Schools all over the world have carried out the educational practice of online learning. In a survey conducted by the Chinese Ministry of Education (2020), as of May 8, 2020, there were 1,454 universities in China conducting online learning, 1.03 million teachers have offered 1.07 million online courses, and a total of 17.75 million college students have participated in online learning. However, inherent problems such as lack of autonomous learning, and insufficient interaction with the learning resources have become increasingly prominent, which may become obstacles restricting the development of online education.

Researchers have carried out a lot of research and discussion around how to produce higher-quality video courses and improve the effectiveness of online education. Among these studies, whether instructors should present and how to present in the online courses is a crucial issue in the field of both research and social practice. The image and voice of instructors in online courses are key elements that must be considered in video course design.

### The Role of Instructor Presence

Instructor presence refers to the presence of multimedia elements such as instructor image and voice in video courses. Based on the different ways of presenting, the current research of instructor presence mainly discussed these multiple dimensions: (1) audiovisual dimension, such as showing only instructor's voice or both the image and voice; (2) time dimension, such as the continuous presence or intermittent presence; (3) fidelity dimension, such as the presence of real instructor image or virtual instructor image; (4) position and ratio dimension, such as the position of the instructor image in the screen; (5) the way of recording, such as embedded and fusion; (6) instructor's demeanor, such as instructor's facial expressions, gestures, eye gazes and etc.

Previous studies have shown that instructor presence had many effects on online learning, including social presence, cognitive load, learning satisfaction, attention, etc. Social presence refers to the degree to which a person is regarded as a “real person” and the degree of perception of connection with others in the process of using media to interact with others (Short et al., 1976). Researchers proposed that, compared with traditional classrooms, online learners lacked an immersive and interactive feeling, which may increase the psychological distance between the learner and the instructor, and adversely affected the learning achievement (Swan, 2003). Presenting the instructor's image or interspersing with social cues, such as gestures, eye gazes, etc., was able to help enhance learner's sense of social presence, stimulate learning motivation and participation, and promote knowledge construction (Richardson and Swan, 2001; Mayer et al., 2003; Dunsworth and Atkinson, 2007).

For the cognitive load, as the individual's cognitive resources are always limited, instructors should effectively utilize learner's cognitive resources to avoid overload, thereby enhancing learning achievement in online learning (Sweller, 1994). As a result, many researchers believe that instructor image is one kind of extra information that can lead to redundant information in the visual channel. When instructor image is presented in the online course videos, it increases extraneous load and hinders the learner's information processing integration (Mayer, 2005; Homer et al., 2008; Kizilcec et al., 2014).

Learning satisfaction is a subjective experience of learners in the learning process, which is often used as a crucial indicator to measure learner's learning situations and the success of teaching (Zhu, 2012). Previous studies have found that the way of the presence of learning materials in online video courses had an impact on learner's learning satisfaction (Zhang et al., 2006). Moreover, Kizilcec et al. (2014) also found that adding instructor's image to course videos was able to prompt learners to produce positive emotional responses, and instructor presence may help increase learner's learning satisfaction.

Furthermore, attention is the orientation and concentration of mental activity to a certain object, which means that attention is a selective cognitive activity. Individuals always selectively pay attention to certain information while ignoring other information accordingly. Psychological studies have found that individuals were highly sensitive to facial information (Gullberg and Holmqvist, 2006). At the same time, facial attention also represents a cultural habit—maintaining eye contact means concentration, interest, and participation (Kendon, 1967; Bavelas et al., 2002). Therefore, theories and eye movement studies have consistently found that the presence of instructors would significantly attract the attention of learners (Choi and Johnson, 2005; Day et al., 2006).

Learner preference is a kind of subjective evaluation for online video courses, which reflects learner's attitudes, emotions, and satisfaction with the courses. It is important for the production and evaluation of online courses. Many studies showed that learners were more inclined to choose online courses with the presence of instructors, thought that these courses were more interesting and were more likely to persist in learning (Kizilcec et al., 2015; Wilson et al., 2018).

From different theoretical perspectives, previous empirical studies did not find consistent effects of instructor presence on online learning. On the one hand, many studies found that instructor presence was able to increase learner's sense of social presence and interest, promote attention investment, stimulate positive emotions, and improve academic performance (Guo et al., 2014; Kizilcec et al., 2014). On the other hand, there were also studies that showed instructor presence did not have a significantly positive impact on online learner's social presence, cognitive load, and academic performance, and may even have a negative impact on learning achievement (Homer et al., 2008). This was partly due to external factors such as experimental design, measurement tools, and teaching style. However, it may also result from the way and quality of instructor presence. According to a survey of 218 MOOC courses from mainstream MOOC platforms worldwide, 94.5% of the course videos showed instructors on the screen, whereas only 5.5% of the videos did not show instructor image at all (Yang et al., 2015). It can be said that, from the perspective of social practice, instructor presence seems to have become the “standard configuration” of current online video courses. Compared with whether instructors should present, the way the presence of instructor may be a more practical issue. Current studies mainly focused on whether instructors were present, as well as the instructor's presence's location, time, instructor's facial expressions, gestures, etc. Less concentration was given to the effect of instructor image and voice on online learning.

### The Role of Instructor's Image and Voice

The presence of an instructor image refers to a person's head, face, neck, and facial features, conveying a large amount of information such as age, gender, health, and emotions. It was found that human's perception of facial attractiveness was very fast (Olson and Marshuetz, 2005). Individuals can quickly perceive and judge differences in facial attractiveness even for visual information that flashed by (13 ms). Attractive images can often trigger people's positive and pleasant emotional experience, prompt people to have a willingness to approach them, and even affect people's judgments of their personality, abilities, and other intrinsic characteristics (Jones et al., 2004), which was also known as “face preference” or “face stereotype” (Dion et al., 1972). Face preference plays an important role in people's mate selection, employment, promotion, and learning as well. Studies have shown that face preference may affect learning by influencing learner's attention, emotion, and motivation.

Previous studies have found that highly attractive faces would attract participant's attention more, produce longer attention spans, and prolong the time it takes for participant's attention to leave and shift to follow-up cognitive tasks (Maner et al., 2007; Sui and Liu, 2009; Leder et al., 2010; Mitrovic et al., 2016). When faces appeared as distractors at the same time as the target cognitive task, highly attractive faces were more likely to produce attentional distractions, cause inhibition of return, and lead to performance degradation (Lindell and Lindell, 2014; Valuch et al., 2015; Hung et al., 2016). When faces were tracked targets, highly attractive faces can promote the distribution and maintenance of attention and improve tracking performance (Liu and Chen, 2012; Li et al., 2016).

Some researchers believe that people's attention to faces may occupy cognitive resources and interfere with other learning tasks, while other researchers propose that learning is a relatively long-term process, and the positive emotions evoked by faces may have a moderating positive effect on learning (Cubukcu, 2013). They can stimulate learner's motivation and promote learning performance (Yang et al., 2014). Westfall et al. (2016) have discussed the influence of instructor's image on learners' learning. They instructed participants to listen to a lecture while watching a photo of an instructor with high or low attractiveness. After the lecture, they completed the task of recognizing the content of the lecture. It was found that participants who watched the photo of the highly attractive instructor performed better than those who watched the photo of an instructor with low attractiveness. As a result, the researchers believed that the positive emotions evoked by the instructor's image positively influenced participants' learning. This has also been confirmed by brain science research. Related studies showed that attractive faces can be used as a reward stimulus to activate the reward system of the observer's brain, such as the nucleus accumbens, amygdala, orbitofrontal cortex, and prefrontal cortex. The release of dopamine in these brain areas will be promoted, which makes individuals feel motivated and happy (Aharon et al., 2001; Kranz and Ishai, 2006; Winston et al., 2007; Cloutier et al., 2008).

Similarly, individuals have preferences for voice (Zuckerman and Driver, 1989). Instructor voice plays an important role in online learning as well. Shoufan (2019) analyzed more than 2,300 “Like” or “Dislike” evaluations of online video courses to investigate the reasons and found that the voice of the instructor was a crucial reason for learners to evaluate the quality of the course. For example, many learners would like a course because the instructor's voice was “confident” and “clear,” whereas some learners would dislike a course as the instructor's voice was “monotonous,” “boring,” or “unclear.” In addition, through research on instructors with pronunciation difficulties, it turned out that any form of voice impairment may affect learner's learning performance (Rogerson and Dodd, 2005).

Some researchers also discussed the influence of computer-synthesized voice on online learning. According to the voice effect theory (Craig and Schroeder, 2017), compared with the voice synthesized by computers, the way of using instructor's voice recording in the video course was more able to promote learner's deep learning and enhance learning performance. Although instructor voice recording is more in line with the learner's preferences, with the development of voice synthesis technology, the difference between computer-synthesized voice and the human voice is rapidly shrinking, and it has broad future development prospects (Chiou et al., 2020).

### The Role of Video and Audio Processing Technology

Technological innovation has brought new changes to instructor presence in online courses. With the support of video beautification, expression capture, voice synthesis, and other emerging technologies, people can conveniently process the image and voice of instructors. This has greatly enriched the way of instructor presence, and its application scenarios are broad. For example, facial expression capture technology and virtual imaging technology allow instructors to project facial expressions, movements, mouth shapes, etc. on virtual characters to replace real people. Moreover, computer voice synthesis technology allows instructors to directly convert the text to voice and add it to video courses without recording by themselves.

Technological innovation will undoubtedly influence future online learning activities. From the perspective of face performance and voice performance, attractive faces and voices can trigger more positive emotions in learners and promote learning. Therefore, it is undoubtedly a positive value to adopt images and voices that learners prefer. On the other hand, different instructor's images and voices will also affect learner's social presence, cognitive load, learning satisfaction, and attention distribution. For example, attractive images and voices may increase the learners' attention to the instructor and reduce their attention to the learning materials and contents. Furthermore, compared with the original image of the instructor, the virtual image may reduce learners' sense of social presence, which in turn affects the learning performance. The new technologies have added many new questions to existing research.

### The Present Study

As a result, this study verified the effects of changes in the image and voice of instructors in online video courses on online learning, including online learner's learning achievement, social presence, learning satisfaction, and achievement-related emotion. And we examined the relationship between learner's evaluation of different ways of instructor image and voice and their learning achievement, social presence, learning satisfaction, and achievement-related emotion. We carried out two simultaneous online experiments. In the first experiment, to examine the effects of the changes in instructor's image on online learning, participants learned a video course with the same content but different images of the same instructor, in one of four conditions: (1) original image, (2) face-beautified image, (3) virtual image, (4) no image. In the second experiment, to explore the impact of the changes in instructor's voice on online learning, participants learned the same video course without the instructor's image, in one of three conditions: (1) original voice, (2) mutated voice, (3) computer-synthesized voice. Based on theories and the results of previous studies, we hypothesized:

1. Compared with learners viewing video courses with instructor image, learners viewing video courses without instructor image would show better learning achievement, lower levels of social presence and satisfaction, and more sense of negative emotion.
2. Compared with learners viewing video courses with the virtual image, learners viewing video courses with the real instructor's image would show better learning achievement, lower levels of social presence, satisfaction, and evaluation, and more sense of negative emotion.
3. Compared with learners viewing video courses with the instructor's original image, learners viewing video courses with the face-beautified image would show lower learning achievement, higher levels of satisfaction and evaluation, and more sense of positive emotion.
4. Compared with learners viewing video courses with the instructor's voice, learners viewing video courses with the computer-synthesized voice would show worse learning achievement, lower levels of social presence, satisfaction and evaluation, and more sense of negative emotion.
5. Compared with learners viewing video courses with the instructor's original voice, learners viewing video courses with the mutated voice would show higher levels of satisfaction and evaluation, and more sense of positive emotion.

## Method

### Participants and Design

In the image experiment, college students from 31 universities in China (*N* = 122, 60 males and 62 females) aged 17–28 years (*M*_age_ = 23.38, *SD*_age_ = 2.4) were recruited and randomly assigned to learn one of the four course versions, including original image (*N* = 32), face-beautified image (*N* = 30), virtual image (*N* = 29), and no image (*N* = 31). None of the students reported having prior knowledge about the content presented in the course.

In the voice experiment, we recruited 93 college students (55 males and 38 females) from 17 Chinese universities aged 18–27 years (*M*_age_ = 23.32, *SD*_age_ = 2.12), who were randomly assigned to study using one of the three course versions, including original voice, mutated voice, and computer-synthesized voice. There were 31 participants in each condition. One thing worth mentioning is that students in the no image group in the image experiment and the original voice in the voice experiment were the same. All the students reported no prior knowledge about the learning content.

Based on the information that they provided in the basic information questionnaire, all participants had normal vision and hearing. They all provided written informed consent and received five dollars for participating in this experiment.

### Materials

#### Video Courses

The video courses were recorded for this study, which lasted about 9 min. The learning content was based on the Chinese national-level MOOC “*Paleography*” describing the meaning, origin, and related allusions of paleography. The learning content and course videos were reviewed by experts to ensure that the content was correct and the difficulty was moderate. The content, slides, duration, and speed of each version were consistent, whereas the forms of instructor presence were different.

**Figure 1:**
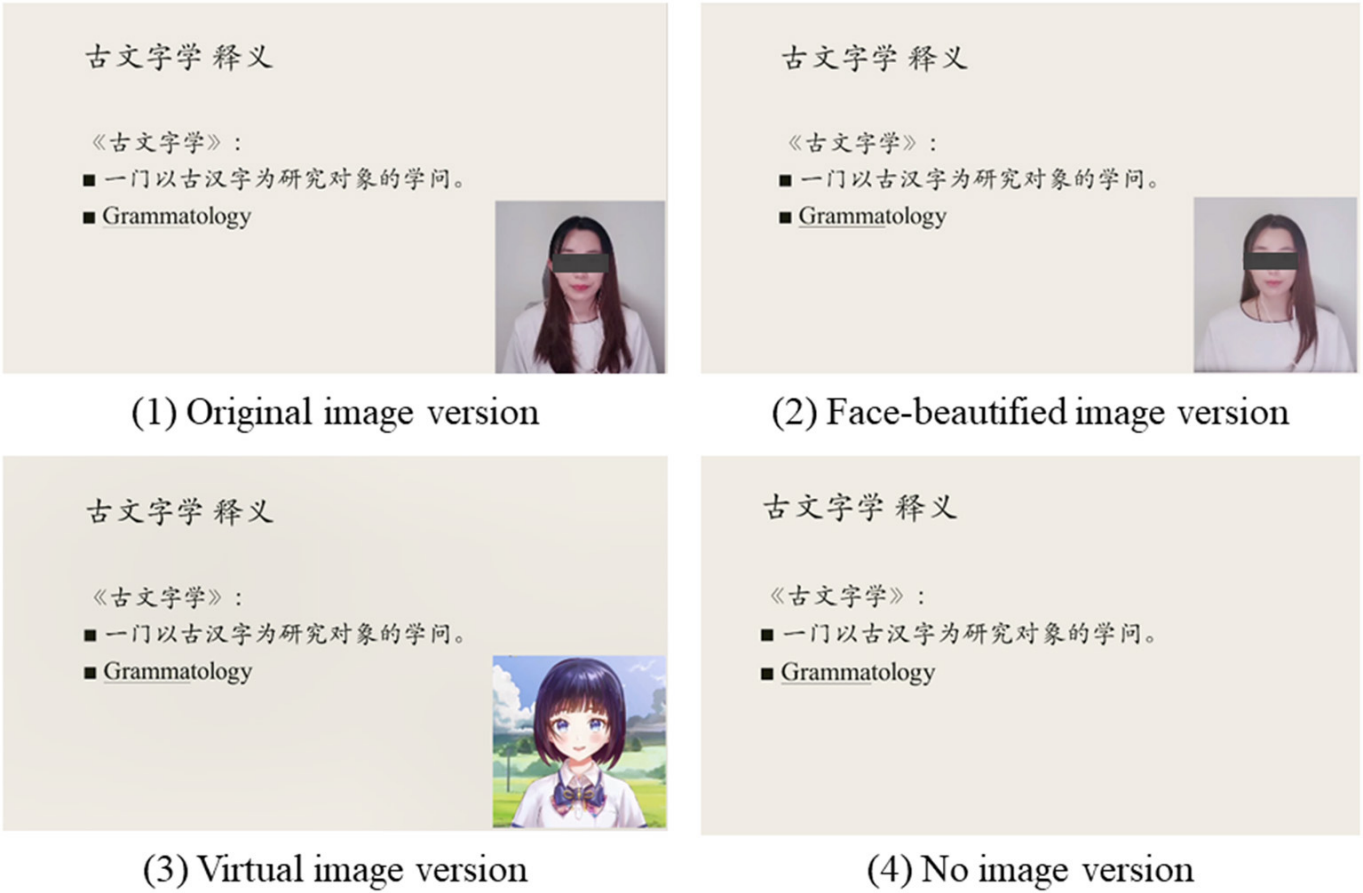
Screen shots of the four versions in the image experiment (after privacy treatment).

For the image experiment, there were four conditions with the instructor's original voice as shown in Figure 1. (1) Original image: the original image of the female instructor was presented. (2) Face-beautified image: the instructor's image was treated with lightening and smoothing of the skin. (3) Virtual image: the instructor's image was replaced by a female 2D cartoon virtual image. The virtual image would follow the changes in the face of the real instructor to make corresponding mouth shapes and expressions. (4) No image: there were only the slides on the screen without the instructor image.

For the voice experiment, the video courses were processed into three versions without the instructor image. (1) Original voice: this version was the same as the no image version in the image experiment. (2) Mutated voice: the voice has been transposed, and we appropriately raised the teacher's pitch (about 1 chromatic scale). According to related research on voice preference (Feinberg et al., 2008; Fraccaro et al., 2013), within a certain range, the attractiveness of female voices with higher pitch is also relatively higher. (3) Computer-synthesized voice: the computer-synthesized voice provided by the iFlytek dubbing platform was adopted. The voice had been used more than 4.5 million times on-demand on the platform, which was widely used and had good results.

#### Measurements

*The basic information questionnaire* included ten items about the learner's gender, age, grade, physical health, such as abnormal vision or hearing, the learning experience in paleography, etc.

*The prior knowledge test* contained four multiple-choice items (total 4 points) and five true-or-false items (total 5 points) to measure learners' prior knowledge about paleography with a full score of 9. Each multiple-choice item had four answer choices, but only one correct answer (e.g., “In which dynasty did Chinese research atmosphere of paleography form? A. Spring and Autumn and Warring States Period; B. Qin and Han Dynasties; C. Tang and Song Dynasties; D. Ming and Qing Dynasties”). And one example of the true-or-false questions was “Is philology the study of the semantics, grammar, and phonetics of ancient Chinese?” All the items in the test were developed by the researchers and examined by one paleography expert to ensure expert validity. The higher the score on this test indicated a higher degree of prior knowledge. There was no significant difference among the four groups in the image experiment [*F*_(3, 188)_ = 0.46, *p* = 0.71 > 0.05, partial η^2^ = 0.01; Table 1] and across the three groups in the voice experiment [*F*_(2, 90)_ = 0.92, *p* = 0.40 > 0.05, partial η^2^ = 0.02; Table 2].

**Table 1:** Means and standard deviations of all dependent variables for each condition in the image experiment.

**Dependent variable**	**Original image** **(*N* = 32)**	**Face-beautified image** **(*N* = 30)**	**Virtual image** **(*N* = 29)**	**No image** **(*N* = 31)**
Prior knowledge	4.41 (1.88)	4.10 (1.73)	4.52 (1.68)	4.10 (1.62)
Retention	9.78 (2.30)	10.00 (2.96)	9.55 (2.46)	11.39 (1.82)
Transfer	7.13 (1.70)	7.07 (1.62)	6.93 (2.03)	8.10 (1.68)
Social presence	30.72 (8.76)	30.67 (8.43)	29.69 (7.35)	31.23 (8.92)
Satisfaction (T)	24.63 (3.24)	24.30 (3.85)	22.28 (3.74)	23.71 (3.85)
Satisfaction (C)	15.69 (2.35)	15.57 (2.64)	14.55 (2.43)	15.77 (2.36)
Satisfaction (I)	11.44 (1.97)	10.73 (2.70)	9.93 (2.43)	9.84 (2.48)
Satisfaction (E)	12.09 (1.75)	11.83 (2.28)	11.28 (1.89)	12.03 (1.68)
Satisfaction	63.84 (8.18)	62.43 (10.11)	58.03 (8.77)	61.35 (8.90)
Enjoyment	14.31 (3.03)	14.70 (3.03)	14.55 (2.93)	15.84 (2.78)
Boredom	11.88 (4.01)	12.00 (4.46)	11.48 (3.64)	9.16 (3.80)
Image evaluation	11.38 (1.56)	12.30 (1.70)	9.55 (1.99)	–

*T, C, I, and E, respectively, represent the instructor teaching, learning content, instructor-learner interaction, and learing environment and equipment of satisfaction*.

**Table 2:** Means and standard deviations of all dependent variables for each condition in the voice experiment.

**Dependent variable**	**Original voice** **(*N* = 31)**	**Mutated voice** **(*N* = 31)**	**Computer synthesized voice** **(*N* = 31)**
Prior knowledge	4.10 (1.62)	4.58 (1.43)	4.16 (1.53)
Retention	11.39 (1.82)	10.42 (1.96)	11.19 (1.89)
Transfer	8.10 (1.68)	7.48 (2.13)	7.58 (1.89)
Social presence	31.23 (8.92)	33.45 (7.99)	33.52 (9.24)
Satisfaction (T)	23.71 (3.85)	23.23 (3.56)	22.84 (3.87)
Satisfaction (C)	15.77 (2.36)	15.55 (2.69)	15.61 (2.80)
Satisfaction (I)	9.84 (2.48)	9.90 (2.90)	10.68 (2.12)
Satisfaction (E)	12.03 (1.68)	12.03 (1.87)	11.61 (1.84)
Satisfaction	61.35 (8.90)	60.71 (9.97)	60.74 (9.16)
Enjoyment	15.84 (2.78)	15.39 (3.23)	15.32 (2.80)
Boredom	9.16 (3.80)	10.48 (3.92)	10.29 (4.16)
Voice evaluation	11.06 (2.25)	10.71 (2.65)	9.39 (1.99)

*T, C, I, and E, respectively, represent the instructor teaching, learning content, instructor-learner interaction, and learing environment and equipment of satisfaction*.

*The learning performance test:* assessed the learners' mastery of the knowledge described in the video course after watching the course video, including a retention test and a transfer test. *The retention test* included 10 multiple-choice items (total 10 points) and five true-or-false items (total 5 points) with a total score of 15 to test learners' retention of key concepts in the video course. For the multiple-choice questions, learners needed to choose one correct answer from four answer choices (e.g., “What is the original meaning of Chinese character ‘zhi’? A. Rest; B. Sunset; C. Stop; D. Toes”). And one example of the true-or-false question was “Is philology an auxiliary tool for people to study history?” The transfer test included eight multiple-choice items with one correct answer (total 8 points) and two multiple-choice items with more than one correct answer (total 2 points) with a total score of 10 to measure learners' ability to transfer the knowledge learned from the video course to solve problems not taught in the course. For example, one of the questions with one correct answer was “Which Chinese character in modern Chinese corresponds to a certain character in ancient Chinese?” whereas one of the questions with more than one correct answer was “Which of the following Chinese characters belong to the category of ‘characters’ in ancient Chinese?” All the items in both tests were developed by the researchers and examined by one paleography expert to ensure expert validity. The higher the score on both tests indicated a higher degree of knowledge retention or knowledge transfer.

*Social presence questionnaire:* A Chinese revised version of the social presence questionnaire developed by Kim and Biocca (1997) was adopted in this study to measure learner's social presence. The questionnaire used a 7-point Likert scale ranging from 1 (totally disagree) to 7 (totally agree) and consisted of eight items. The odd-numbered items were scored positively, while the even-numbered items were scored in reverse. The final score of this scale was the sum of ratings for each item. One example of the item was “When the video ended, I felt like I had returned to the real world from a trip.” The social presence questionnaire showed high internal consistency (Cronbach's α = 0.83). The higher the score on this scale meant a higher level of social presence.

*Learning satisfaction questionnaire:* was from the video course learning satisfaction questionnaire revised by Yang (2014). There were four factors, including instructor teaching (total 6 items), learning content (total 5 items), instructor-learner interaction (total 3 items), learning environment and equipment (total 3 items), and 17 items on a 5-point scale in the questionnaire. The final score of each factor was the sum of ratings for each item, and the final score of this scale was the sum of scores of all the factors. Examples of items in each factor included “The instructor is serious in class and cares about learners' learning”, “The content in the video attracts me and helps me”, “Through video learning, I can fully participate in the learning process”, and “I am satisfied with the normal operation of the video course”. The learning satisfaction questionnaire showed moderate-to-high internal consistency (instructor teaching Cronbach's α = 0.87; learning content Cronbach's α = 0.79; instructor-learner interaction Cronbach's α = 0.78; learning environment and equipment Cronbach's α = 0.81). The higher the score on each factor meant a higher level of satisfaction.

*Achievement-related emotion questionnaire:* was from the online learning achievement-related emotion questionnaire of Artino and Jones (2012), which was able to measure learner's emotional levels of enjoyment and boredom in online learning. It consisted of two factors (enjoyment and boredom), 9 questions, on a 5-point scale. There were four items in the enjoyment factor and five items in the boredom factor. The final score of each factor was the sum of ratings for each item. Examples of the items in each factor included “I enjoy studying the course” and “I would rather do something else than study the course.” The internal consistency of the achievement-related emotion questionnaire was high (enjoyment Cronbach's α = 0.86; boredom Cronbach's α = 0.90). The higher score on the two factors meant higher levels of enjoyment and boredom, respectively.

*Instructor's image/voice evaluation questionnaire:* In order to investigate learner's evaluation of instructor's image/voice, evaluation questionnaires were developed by the researcher. Both the instructor's image evaluation questionnaire and voice evaluation questionnaire included two items and used a 7-point Likert scale. The final score of each scale was the sum of ratings for each item, with higher score indicating a higher level of evaluation. The image evaluation items consisted of “I think the instructor looks decent and generous” and “I think the instructor has a good image and makes me feel comfortable.” And the voice evaluation items included “The instructor speaks clearly, expresses fluently, and speaks at a moderate speed” and “I think the instructor's voice is mellow and attractive.” The instructor's image evaluation questionnaire showed high internal consistency (Cronbach's α = 0.87) and the instructor's voice evaluation questionnaire showed moderate internal consistency (Cronbach's α = 0.70).

### Procedure

**Figure 2:**
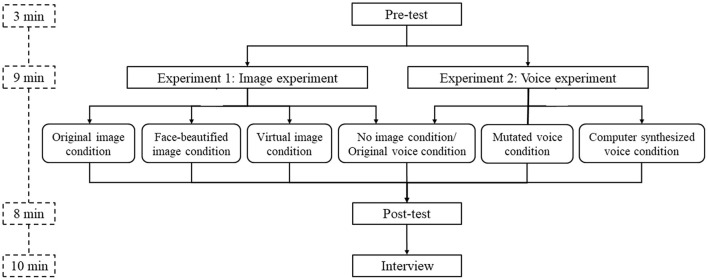
Diagram of experiment design.

The study was conducted online and in individual sessions of ~30 min. Two experiments including the image experiment and voice experiment were carried out simultaneously. As shown in Figure 2, all the learners first completed the pre-test, consisting of the basic information questionnaire and the prior knowledge questionnaire. All the learners first completed the pre-test, consisting of the basic information questionnaire and the prior knowledge questionnaire. Then they were randomly assigned to one of the conditions, given a link to the video course, and instructed to use a laptop and wear headphones to watch the video individually within the specified time. Immediately after finishing the video course, learners took the post-test, including the learning performance test, social presence questionnaire, learning satisfaction questionnaire, achievement-related emotion questionnaire, and instructor's image/voice evaluation questionnaire. Finally, semi-structured interviews with learners were conducted to collect their feedback on their learning experience.

## Results

**Figure 3:**
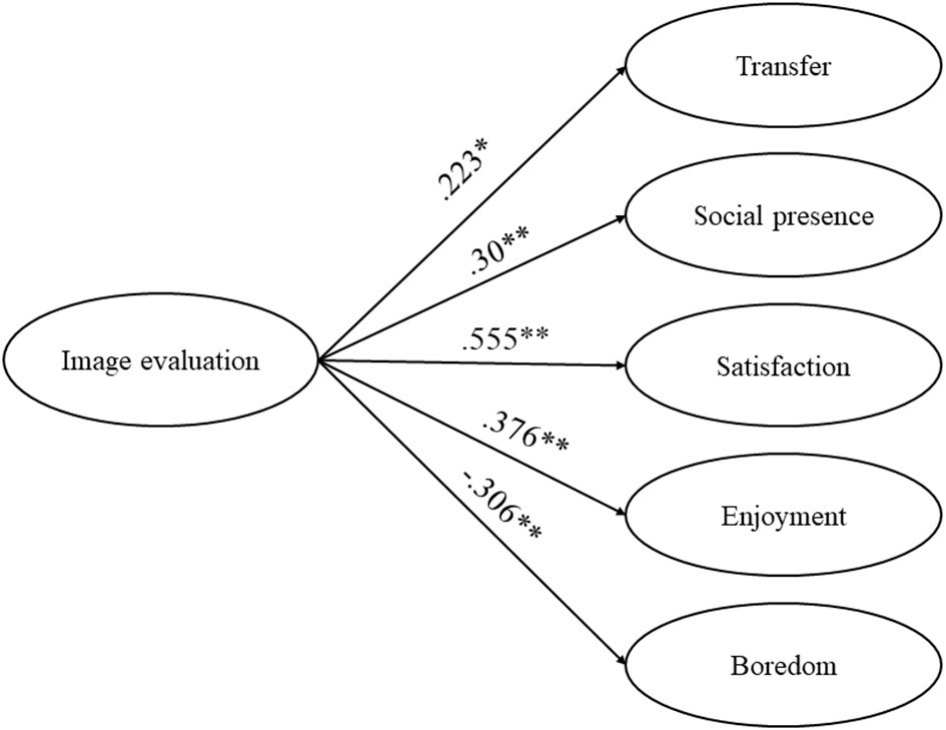
Results of the linear regression of image evaluation on each variable.

**Figure 4:**
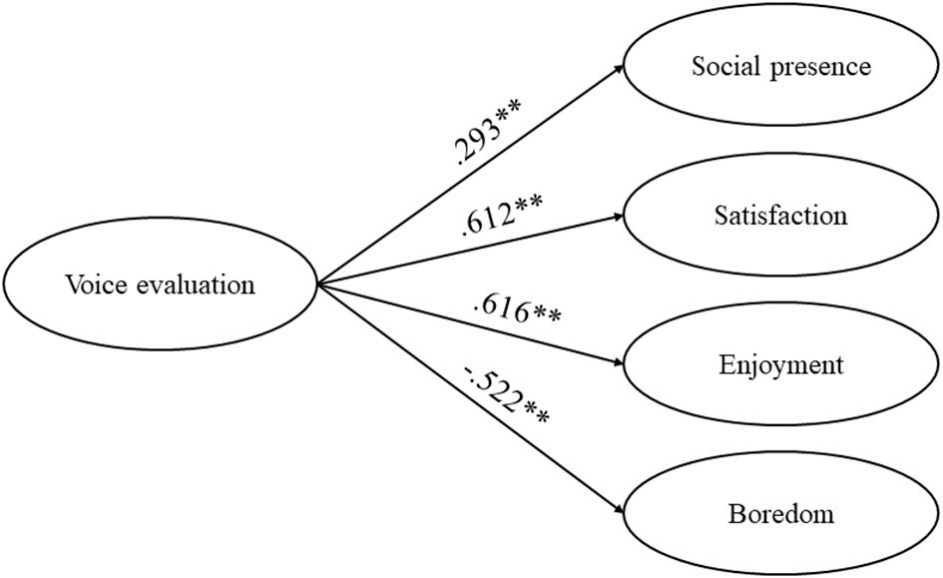
Results of the linear regression of voice evaluation on each variable.

To compare the differences in learning performance, including retention and transfer, of the four experimental groups in the image experiment and the three experimental groups in the voice experiment, we conducted two analyses of covariance (ANCOVAs), with the prior knowledge as the covariance, the conditions as the independent variables, and the retention scores or the transfer scores as the dependent variables. The reason that the ANCOVA method was chosen for this analysis and the prior knowledge was used as the covariance was that, although we did not find a statistically significant difference in the pre-test among the four groups in the image experiment and across the three groups in the voice experiment, there were differences in the pre-test scores among the groups in both experiments, and the ANCOVA method was able to eliminate the possible unwanted variance on the dependent variable and increased test sensitivity (Tabachnick and Fidell, 2013). Moreover, the one-way analysis of variance (ANOVA) was employed to test differences in social presence, satisfaction, achievement-related emotion, and image evaluation/voice evaluation across the experimental groups in the two experiments. Descriptive statistics (means and standard deviations) of the image experiment and the voice experiment were presented in Tables 1, 2, respectively. Furthermore, two linear regressions were conducted to examine the relationship between learners' evaluation of different ways of instructor presence and other variables to further understand how the changes in the image and voice of instructors influenced online learning. The results of the correlation coefficients were present in Tables 3, 4, and the results of the linear regression were present in Figures 3, 4. In addition, interviews were transcribed and analyzed to further understand the results found in this study.

**Table 3:** Correlation coefficients of each variable and image evaluation (*N* = 122).

**Variable**	**Image evaluation**	
	**Correlation**	***p***
Retention	0.117	0.268
Transfer	0.223^*^	0.034
Social presence	0.300^**^	0.004
Satisfaction	0.555^**^	0.000
Enjoyment	0.376^**^	0.000
Boredom	−0.306^**^	0.003

* *p < 0.05*,

** *p < 0.01*.

**Table 4:** Correlation coefficients of each variable and voice evaluation.

**Variable**	**Voice evaluation**	
	**Correlation**	***p***
Retention	0.095	0.368
Transfer	0.078	0.455
Social presence	0.299^**^	0.004
Satisfaction	0.612^**^	0.000
Enjoyment	0.616^**^	0.000
Boredom	−0.522^**^	0.000

* *p < 0.05*,

** *p < 0.01*.

### The Effects of Instructor's Image on Online Learning

#### Learning Performance

ANCOVA showed that there was a significant difference on retention and transfer across the four groups, *F*_(3, 117)_ = 4.187, *p* = 0.007 < 0.01, partial η^2^ = 0.097; *F*_(3, 117)_ = 2.933, *p* = 0.036 < 0.05, partial η^2^ = 0.07, respectively. As predicted in the first hypothesis, *post-hoc* LSD tests on retention and transfer found that the no image group showed significantly higher performance than the original image group, respectively, *MD* = 1.61, *p* = 0.009 < 0.01; *MD* = 0.97, *p* = 0.031 < 0.05, the face-beautified image group, respectively, *MD* = 1.39, *p* = 0.027 < 0.05; *MD* = 1.03, *p* = 0.024 < 0.05, and virtual image group, respectively, *MD* = 1.84, *p* = 0.004 < 0.01; *MD* = 1.17, *p* = 0.012 < 0.05, whereas the other group comparisons showed no significant difference both on retention and transfer.

The above results indicated that learners who used the video course without the instructor's image benefited more than learners who viewed the video course with the instructor's image. Moreover, the ways of the presence of instructor image did not influence online learning.

#### Social Presence

The ANOVA on social presence showed no significant difference among the four groups, *F*_(3, 118)_ = 0.173, *p* =0.914 > 0.05, partial η^2^ = 0.004, indicating that instructor image presence had no significant impact on online learner's social presence.

#### Satisfaction

We conducted five ANOVAs to examine the difference in satisfaction across four groups, with the instructor teaching satisfaction, learning content satisfaction, instructor-learner interaction satisfaction, learning environment and equipment satisfaction, and the whole satisfaction as the dependent variables, respectively. The results showed that there was a significant difference in instructor-learner interaction satisfaction across the four groups, *F*_(3, 118)_ = 3.04, *p* = 0.032 < 0.05, partial η^2^ = 0.072. As predicted in the first and third hypotheses, post hot LSD tests revealed that the original image group had significantly higher instructor-learner interaction satisfaction than the virtual image group, *MD* = 1.51, *p* = 0.016 < 0.05, and the no image group, *MD* = 1.60, *p* = 0.009 < 0.01.

Although no significant difference in instructor teaching satisfaction and the whole satisfaction was found across the four groups, *F*_(3, 118)_ = 2.388, *p* = 0.072 > 0.05, partial η^2^ = 0.057; *F*_(3, 118)_ = 2.265, *p* = 0.085 > 0.05, partial η^2^ = 0.054, respectively, the virtual image group showed significantly lower instructor teaching satisfaction than the original image group, *MD* = −2.35, *p* = 0.013 < 0.05, and the face-beautified image group, *MD* = −2.02, *p* =0.034 < 0.05, and the virtual image group showed significantly lower satisfaction than the original image group, *MD* = −5.81, *p* = 0.014 < 0.05.

These results suggested that, in online learning, compared with the video courses without the instructor's image, the video courses with the original image of the instructor were able to enhance the learner's sense of instructor-learner interaction. In addition, compared with the video courses with the real instructor's image, the use of the virtual instructor's image would reduce learners' satisfaction with instructor-learner interaction and instructor teaching.

#### Achievement-Related Emotion

We conducted two ANOVAs to investigate the effects of instructor image on online learner's achievement-related emotion, with enjoyment and boredom as the dependent variables, respectively. The ANOVA on boredom showed significant differences across the four groups, *F*_(3, 118)_ = 3.434, *p* = 0.019 < 0.05, partial η^2^ = 0.08. *Post-hoc* LSD tests revealed that, of the four groups, the no image group reported lower levels of boredom than the original image group, the face-beautified image group, and the virtual image group (respectively, *MD* = −2.71, *p* = 0.008 < 0.01; *MD* = −2.84, *p* = 0.006 < 0.01; *MD* = −2.32, *p* = 0.026 < 0.05), indicating that the presence of instructor image had caused the learner's emotion of boredom.

#### Image Evaluation

The ANOVA results showed a significant difference in image evaluation across the original image group, the face-beautified image group, and the virtual image group, *F*_(2,88)_ = 18.696, *p* = 0.000 < 0.01, partial η^2^ = 0.298. As predicted in the third hypothesis, the results of *post-hoc* LSD tests indicated that the learner's evaluation of the virtual image was significantly lower than the original image and the face-beautified image (respectively, *MD* = −1.82, *p* = 0.000 < 0.01; *MD* = −2.75, *p* = 0.000 < 0.01). Moreover, as predicted in the second hypothesis, their evaluation of the face-beautified image was significantly higher than the original image, *MD* = 0.93, *p* = 0.041 < 0.05.

These results suggested that, compared with the virtual cartoon image, online learners preferred the image of the real instructor. Furthermore, the video beautification had a certain effect on improving the image of real instructors.

To further explore the relationship between image evaluation and other variables, respectively, five linear regression analyses were conducted. The correlation results for each variable were shown in Table 3. Among these variables, image evaluation had a statistically significant positive relationship with transfer (*r* = 0.223), social presence (*r* = 0.3), satisfaction (*r* = 0.555), and enjoyment (*r* = 0.376), whereas it had a statistically significant negative relationship with boredom (*r* = −0.306). As a result, these five variables were included in the linear regression analyses, with the image evaluation as the independent variable, and the five variables as the dependent variable, respectively in the five analyses.

As shown in Figure 3, the statistically significantly positive effects of image evaluation on transfer (β = 0.223, *B* = 0.191, *p* = 0.034 < 0.05, *R*^2^ = 0.05), social presence (β = 0.30, *B* = 1.18, *p* = 0.004 < 0.01, *R*^2^ = 0.09), satisfaction (β = 0.555, *B* = 2.487, *p* = 0.000 < 0.01, *R*^2^ = 0.309), and enjoyment (β = 0.376, *B* = 0.54, *p* = 0.000 < 0.01, *R*^2^ = 0.141) were all significant, while image evaluation was the independent variable that significantly negatively predicted boredom (β = −0.306, *B* = −0.593, *p* = 0.003 < 0.01, *R*^2^ = 0.094).

The above results showed that learner's better evaluation of instructor image was able to predict higher transfer score, more sense of social presence and enjoyment, and higher satisfaction of the course, whereas learners who had higher image evaluation had less sense of boredom.

### The Effects of Instructor's Voice on Online Learning

#### Learning Performance

ANCOVA did not find a significant effect of conditions on retention and transfer across the three voice group, respectively, *F*_(2, 89)_ = 2.818, *p* = 0.065 > 0.05, partial η^2^ = 0.06; *F*_(2, 89)_ = 1.075, *p* = 0.346 > 0.05, partial η^2^ = 0.024, suggesting that the changes in the voice of the instructor did not affect online learner's learning performance.

#### Social Presence

The results of ANOVA showed that there was no significant difference in social presence among the three groups, *F*_(2, 90)_ = 0.112, *p* = 0.894 > 0.05, partial η^2^ = 0.002, indicating that different ways of instructor's voice in online video courses did not influence online learner's sense of social presence.

#### Satisfaction

No significant effect of instructor voice on instructor teaching satisfaction, learning content satisfaction, instructor-learner interaction satisfaction, learning environment and equipment satisfaction, and the whole satisfaction was found in the results of ANOVA, suggesting that the instructor's voice did not affect online learner's satisfaction of the course.

#### Achievement-Related Emotion

There was no significant difference in enjoyment and boredom across the three groups, respectively, *F*_(2, 90)_ = 0.283, *p* = 0.754 > 0.05, partial η^2^ = 0.006; *F*_(2, 90)_ = 1.007, *p* = 0.37 > 0.05, partial η^2^ = 0.022, showing that these three groups were not different on sense of enjoyment and boredom.

#### Voice Evaluation

The ANOVA on voice evaluation showed a significant difference in voice evaluation of the three voice course versions, *F*_(2, 90)_ = 4.527, *p* = 0.013 < 0.05, partial η^2^ = 0.091. As predicted in the fourth hypothesis, learner's evaluation of computer-synthesized voice was significantly lower than the evaluations of original voice and mutated voice, respectively, *MD* = −1.68, *p* = 0.005 < 0.01; *MD* = −1.32, *p* = 0.027 < 0.05, while there was no significant difference in voice evaluation between the original voice group and the mutated voice group.

These results suggested that online learners had a lower evaluation of computer-synthesized voice than the voice of a real teacher. In addition, the voice changing system did not significantly enhance the instructor's voice.

We conducted four linear regression analyses to examine the relationship between voice evaluation and other variables, respectively. According to Table 4, voice evaluation had a significant positive relationship with social presence (*r* = 0.299), satisfaction (*r* = 0.612), and enjoyment (*r* = 0.616), while it had a statistically significant negative relationship with boredom (*r* = −0.522). Thus, we included the four variables in the linear regression analyses, with the voice evaluation as the independent variable, and the four variables as the dependent variable, respectively in the four analyses.

Based on Figure 4, the significantly positive effects of voice evaluation on social presence (β = 0.293, *B* = 1.062, *p* = 0.004 < 0.01, *R*^2^ = 0.086), satisfaction (β = 0.612, *B* = 2.359, *p* = 0.000 < 0.01, *R*^2^ = 0.374), and enjoyment (β = 0.616, *B* = 0.75, *p* = 0.000 < 0.01, *R*^2^ = 0.379) were all significant, while voice evaluation was the independent variable that significantly negatively predicted boredom (β = −0.522, *B* = −0.863, *p* = 0.000 < 0.01, *R*^2^ = 0.273).

These results showed that learners with higher voice evaluation had more sense of social presence and enjoyment, and were more satisfied with the course, while learners with higher voice evaluation had less sense of boredom.

## Discussion and Conclusion

This study investigated the effects of the changes in the image and voice of instructors in online video courses on online learning. We carried out two online experiments simultaneously. In the first experiment, we examined the effects of the changes in instructor's image on online learner's learning achievement, social presence, learning satisfaction, and academic emotion, and examined the relationship between learners' evaluation and their learning achievement, social presence, learning satisfaction, and academic emotion. In the second experiment, we explored the impact of the changes in instructor's voice on online learner's learning achievement, social presence, learning satisfaction, and academic emotion, and examined the relationship between learners' evaluation and these variables. The findings are discussed below.

First, learners viewing the no image course version showed significantly higher learning achievements and less academic boredom. One possible explanation is that the instructor image attracts more attention of learners, which adds extraneous cognitive load and interferes with the cognitive processing activities of learners (Mayer, 2005; Day et al., 2006). For example, some learners (10.3%) reported in the interview that “*The cartoon image of the virtual instructor was very novel and cute. I couldn't help but stare at the image during the learning process. As a result, I missed a lot of knowledge*.” At the same time, the learner's learning motivation may also play a role. As the subjects were all college students who had already known the topic of the course through the recruitment information in advance, they may all have a high level of interest in the course topic and learning motivation. For example, most learners (77.2%) expressed their interest in learning the content of this course in the interview. Therefore, in the learning process, there may be conflicts between their subjective learning motivation and objective cognitive interference, which induces learner's negative emotions (Kizilcec et al., 2014). However, the presence of instructor image also had a certain positive impact on online learning.

Both the two ways of the real instructor image's presence can significantly promote learners' learning satisfaction of instructor-student interaction. The reason may be that the presence of a real instructor's image enables learners to directly observe the instructor's appearance, facial expressions, gestures, etc., and gives students a sense of interaction and communication with the instructor (Dunsworth and Atkinson, 2007). In the interview, some subjects (22.6%) viewing the video courses without instructor image pointed out that “*There was no interactive content involved in the course learning*” and “*It felt like watching slides instead of taking a course with an instructor*.”

Second, learners evaluated the two kinds of the real instructor image significantly better than the virtual one. Meanwhile, the two kinds of real instructor image led to significantly higher learning satisfaction of instructor-student interaction and instructor teaching. This may be related to the maturity of the image processing technologies, the design of virtual instructor image, learner's personal preference, and learner's perception of the instructor (Short et al., 1976; Jones et al., 2004). Although the current facial expression capture technology can make the avatar follow the person's facial changes to make corresponding mouth shapes, expressions, blinking movements, etc., it is lacking in sensitivity and expressiveness. The virtual image still cannot fully restore the demeanor of a real teacher. And the avatar cannot further convey more complex emotional signals such as eye expressions and micro-expressions. Moreover, there are various types of virtual images. As learners have different evaluations of different teachers, different virtual images may also affect learner's perception and evaluation. This study used a 2D cartoon girl image as the instructor's image. Compared with the 3D realistic style character image, the 2D image may make learners feel a stronger sense of virtuality and weaker simulation. At the same time, the image of a younger girl may also give learners the impression of low qualifications and weak teaching ability, thereby reducing learners' learning satisfaction.

Furthermore, combining the interview data, we found that the learners' personal preferences also played an important role in virtual image group students' learning. Some subjects (13.8%) preferred the image of the anime style, thus they expressed more like for the virtual instructor and that they would pay more attention to the virtual instructor during the learning process. However, some subjects (37.9%) had low acceptance of cartoon images. They hardly paid attention to the virtual teacher during the learning process and tended to make a lower evaluation of the virtual instructor image. In addition, the subjects did not know the way the virtual image was generated, which was based on the real instructor. In the interview, many subjects (44.8%) said that they did not perceive the virtual image as a human teacher, but as an auxiliary teaching agent. This difference in perception may also affect learners' evaluation of instructor image and learning satisfaction.

However, there was no significant difference in learning achievement between the virtual image group and the two instructor image groups. Although the image of the virtual instructor is still inferior to the image of the real instructor in the overall evaluation, it will not have a significant negative impact on the learning effect. In the future, we can flexibly choose different virtual images to appear on the video according to the teaching needs, but we should pay special attention to the need to choose the appropriate image according to the preference of learners to better support online learning.

Third, compared with the instructor's original image, the application of the face-beautified image was able to significantly improve learners' evaluation of the image, but it did not show an obvious effect on learning achievement, satisfaction, and achievement-related emotion. This shows that video beautification technology has a certain effect on improving the image of teachers, but it is not a core factor that affects the learning process and effect. The educational value of video beautification, virtual image, and other technologies may not be reflected in the direct promotion of learning, but more as an auxiliary tool for video design.

In the dimension of social presence, no significant difference was found across the experimental groups. This shows that, in order to truly enhance the learners' sense of social presence, it is not enough to simply add the instructor's image to the video. The video needs to be designed well and meticulously, such as flexibly presenting instructor images according to the needs of teaching activities. For example, when the learner needs to pay attention to the instructor, the instructor's image should be presented; when the learner needs to pay attention to the teaching materials, the instructor's image should be hidden.

Furthermore, it was found that image evaluation had a significant positive relationship with transfer, social presence, satisfaction, and enjoyment, whereas it had a significant negative relationship with boredom. This can partly be explained by face preference, that is, attractive faces can trigger learner's positive emotions and contribute to the learning process (Dion et al., 1972; Cloutier et al., 2008; Yang et al., 2014). Therefore, in the process of video production, it is necessary to notice that the image of the instructor is not the core factor that affects the learning process and effect, but also to realize that the image of the instructor has an important influence on the psychological feelings of learners, such as the emotions of the learners. We need to comprehensively use image processing technology in practice to show a good instructor image as much as possible.

Fourth, the evaluations of both the original voice and the mutated voice by students were significantly higher than the computer-synthesized voice, but there was no significant difference in learning achievement, social presence, satisfaction, and achievement-related emotion between the computer-synthesized voice group and the real instructor voice groups. In the interview, only a few subjects (6.5%) clearly stated that the instructor's voice was like artificial intelligence robots. Most of the subjects (51.6%) only felt slightly strange, such as the unusual pronunciation of individual words of the instructor, but they were not sure or were unexpected that the instructor's voice was not from a real person. The rest of the subjects had no special perception of the computer-synthesized voice. This finding indicates that with the advancement of voice synthesis technology, the quality of computer-synthesized voice is gradually approaching the voice of real instructors. Although psychologically learners still tend to prefer real instructors' voices, it is increasingly difficult for them to tell the differences between the real instructor's voice and the computer-synthesized voice (Chiou et al., 2020). Therefore, in the future, it may become a new trend to use computer voice synthesis technology to replace real instructor dubbing, and to design and produce learning materials quickly, conveniently, and at low cost.

Finally, compared with the instructor's original voice, we did not find the effect of mutated voice on satisfaction, achievement-related emotion, and evaluation. This may be related to the design and production of video materials. In the production process of the video material, we only performed a slight pitch shifting process on the original sound's pitch (increased by about 1 chromatic scale). This simple and slight process made the difference between the two sounds relatively limited, showing no obvious impact on learners' perception of the voice and learning process. Moreover, we processed it to raise the pitch instead of lowering it. This is based on the related research of voice preference showing that, within a certain range, the pitch of the female voice has a positive relationship with its attractiveness level (Fraccaro et al., 2013). However, its influence on the learning process has not been confirmed by empirical studies. Therefore, this study provides evidence that the pitch of female instructor voice in online video courses does not influence online learning.

In the interview, some subjects (35.5%) also pointed out that “*I felt the instructor's voice very young*.” In addition, it was found that changes in pitch may affect learners' perception of the instructor's speaking speed and the effect of information reception. In the mutated voice group, nearly half of the subjects (48.4%) reported, “*The instructor's speaking speed was too fast, and many knowledge points passed quickly*.” In fact, we only changed the pitch of the instructor's voice, and all other aspects, including the instructor's speaking speed, were not changed. Learners in other experimental groups did not report the same thing. As a result, we believe that the changes in the pitch of the instructor's voice may be a potential research topic. For example, some research found that people felt lower-pitched voices more leadership and prestige (Anderson and Klofstad, 2012; Klofstad et al., 2012; Tigue et al., 2012). Thus, raising the pitch of the instructor's voice may not be an appropriate method to increase the attractiveness of the voice. On the contrary, it may reduce the learner's judgment of the instructor's competence. Proper pitch reduction of the instructor's voice may make the sound more calm, clear, and convincing, thereby enhancing learning.

In addition, the evaluation of the instructor's voice positively predicted social presence, learning satisfaction, and enjoyment, but negatively predicted boredom. This result partially supports the voice preference (Zuckerman and Driver, 1989; Shoufan, 2019). Based on this, we should pay attention to the in-depth research and application of voice synthesis technology to reduce the cost and technical threshold of the production of learning materials. Moreover, we need to study the voice characteristics that can promote the information reception and processing, and use this to improve the quality of voices in course videos and learning resources.

## Limitations and Future Research

The following limitations to the current study should be considered. Firstly, we did not measure the learner's attention to the video course, which is an important factor influencing the learning process (Pi et al., 2019). Learner's attention can be examined by eye movements metrics, such as the mean fixation duration, dwell time over the AOI, the ratio of pupil size change, etc., which can reflect learner's participation and cognitive load (Zu et al., 2019). Future research needs to combine eye movement analysis to enhance the objectivity of data collection. Secondly, we conducted online experiments which may lead to unknown or uncontrollable influences on the learning process during the experiments. For example, due to a lack of external constraints, learners may be too relaxed and lax, which affects the credibility of the collected data. Thirdly, in terms of the experimental material design, this study utilized 9-min video materials, which were shorter than the 40-min traditional class materials. Moreover, we only designed single-sex instructors and did not consider the possible impact of instructor's gender differences on learning (Valuch et al., 2015). The instructor's image conveys a lot of information, including gender. Researchers believe that averageness, symmetry, and sexual dimorphic features are the three main factors affecting facial attractiveness (Rhodes, 2006). Among these factors, sexual dimorphic features are the characteristics of masculine or femininity. People prefer images of their favorite gender (Mitrovic et al., 2016). As a result, learners may have different evaluations and perceptions of instructors of the same sex or the opposite sex. In addition, there were differences in the instructor's clothes and hairstyles between the real image groups and the virtual image group. Future research needs to further explore the effects of instructor's gender on learning and keep instructor's clothes and hairstyles the same in different conditions. Finally, this study only recruited college students as the participants in experiments and did not recruit younger learners such as elementary and middle school students. College students' learning habits are more mature, who may be less sensitive to changes in the image and voice of instructors compared with younger learners. Future research needs to be conducted to generalize the results of this study by recruiting learners of different ages.

With the rapid development of online education, instructor presence research will also continue to deepen. Combining the findings and limitations of this research, we believe that there are three points that can be the focus of future research. Firstly, this study focused on online learners. However, it is also worth studying that how instructors, as the main body of presence, view the image presence and what impact this may have on their teaching attitudes and behaviors. For example, will the presence of instructor image increase the pressure on instructors who lack experience in online teaching? Will the application of video beautification or virtual image technology help promote teachers' self-confidence and ease the discomfort when facing the camera? Secondly, this study pays more attention to the measurement of indicators directly related to learning, but there are also some external factors that may also have an important impact on online teaching and learning. For example, in online teaching or communication, we often tend to encourage students to turn on the camera to enhance the sense of interaction. However, due to various reasons, learner's willingness to turn on the camera is generally low. The use of virtual images and other technologies may be able to encourage learners to lay down their psychological burdens, increase their willingness to participate in the interaction, and promote the improvement of learning effects. Finally, there are many ways to process both the image and the voice. This study only investigated the impact of the 2D cartoon image and the slight rise of instructor voice pitch on learning. More research needs to be conducted to explore what kind of image or voice is most suitable for online learning, and what preferences do learners of different ages, genders, and majors have? Future research is needed to provide a reference for more scientific and personalized curriculum design.

## Data Availability Statement

The raw data supporting the conclusions of this article will be made available by the authors, without undue reservation.

## Ethics Statement

Ethical review and approval was not required for the study on human participants in accordance with the local legislation and institutional requirements. Written informed consent to participate in this study was provided by the participants' legal guardian/next of kin.

## Author Contributions

MY and JS conceived the study. JS and AW contributed to the supervision. MY conducted the experiment and collected the data. MY and JZ analyzed and interpreted the data and contributed to the writing of the manuscript. All authors have read and agreed to the published version of the manuscript.

## Funding

This work was supported by the Key Education Research Project in 2020 Sponsored by Yuyue Educational Development Foundation in Center for Research on Pre-K12 Education of Peking University (No: JCJYYJ201902).

## Conflict of Interest

The authors declare that the research was conducted in the absence of any commercial or financial relationships that could be construed as a potential conflict of interest.

## Publisher's Note

All claims expressed in this article are solely those of the authors and do not necessarily represent those of their affiliated organizations, or those of the publisher, the editors and the reviewers. Any product that may be evaluated in this article, or claim that may be made by its manufacturer, is not guaranteed or endorsed by the publisher.
